# Evaluation of a Novel Precordial Lead System for the Electrocardiographic Diagnosis of Right Ventricular Enlargement in Dogs

**DOI:** 10.3390/vetsci9080399

**Published:** 2022-07-30

**Authors:** Giovanni Grosso, Tommaso Vezzosi, Cesara Sofia Pergamo, Martina Bini, Valentina Patata, Oriol Domenech, Rosalba Tognetti

**Affiliations:** 1 Department of Veterinary Sciences, University of Pisa, Via Livornese, San Piero a Grado, 56122 Pisa, Italy; tommaso.vezzosi86@gmail.com (T.V.); ce.pergamo@gmail.com (C.S.P.); rosalba.tognetti@unipi.it (R.T.); 2 Anicura Istituto Veterinario Novara, Strada Provinciale 9, Granozzo con Monticello, 28060 Novara, Italy; binimartina93@gmail.com (M.B.); valentinapatata89l@gmail.com (V.P.); odomenech1973@gmail.com (O.D.)

**Keywords:** electrocardiography, right heart dimensions, 12-lead ECG, cardiology, canine

## Abstract

**Simple Summary:**

Echocardiography is the gold-standard method for the assessment of cardiac chamber enlargement. However, precordial leads can play a complementary role in the non-invasive detection of cardiac remodeling. Therefore, the aim of this study was to evaluate the diagnostic accuracy of precordial leads for the detection of right ventricular enlargement in dogs. Healthy dogs and dogs with echocardiographic right ventricular enlargement were enrolled in this prospective observational study. All the electrocardiographic measurements were assessed in both limb and precordial leads, and their diagnostic accuracy for the detection of right ventricular enlargement was evaluated. A total of 84 dogs, 27 with right ventricular enlargement and 57 controls, were enrolled. Q wave amplitude in lead aVR (cutoff > 0.10 mV), R/S ratio in lead V4 (cutoff < 1.15), R/S ratio in lead V5 (cutoff < 1.95) and S wave amplitude in lead V6 (cutoff > 0.70 mV) showed suitable diagnostic accuracy in detecting right ventricular enlargement. Only nine dogs (33%) with right ventricular enlargement presented a right shift of the mean electrical axis. Differently, 19 out of 27 (70%) showed at least one of the identified precordial lead criteria. Adding the reported precordial leads criteria increases the diagnostic accuracy of electrocardiography for the detection of right ventricular enlargement in dogs.

**Abstract:**

The purpose of this study was to evaluate the reliability of precordial leads for the detection of right ventricular enlargement (RVE) in dogs. This was a prospective observational study. The RVE was defined by echocardiography. The amplitude (mV) of the Q, R, and S waves, the R/S ratio, and the mean electrical axis (MEA) of the QRS complex were assessed on the 12-lead ECG. The ROC curve and the Youden index yielded the best cutoffs for RVE detection. An area under the curve (AUC) > 0.7 defined suitable diagnostic accuracy. A total of 84 dogs, 27 with RVE and 57 healthy controls, were enrolled. Q wave amplitude in aVR (cutoff > 0.10 mV; AUC = 0.727), R/S ratio in V4 (cutoff < 1.15; AUC = 0.842), R/S ratio in V5 (cutoff < 1.95; AUC = 0.839) and S wave amplitude in V6 (cutoff > 0.70 mV; AUC = 0.703) showed suitable diagnostic accuracy in detecting RVE. Among dogs with RVE, only 9/27 (33%) presented a right shift of MEA. Differently, 19/27 (70%) showed at least one of the identified precordial lead criteria. Assessment of the R/S ratio in V4 and V5 and S wave amplitude in V6 increases the diagnostic accuracy of ECG in distinguishing between dogs with RVE and healthy dogs.

## 1. Introduction

Electrocardiography (ECG) represents the gold-standard method for the diagnosis of cardiac arrhythmias at rest in dogs [1,2]. Differently, two-dimensional transthoracic echocardiography represents the gold-standard non-invasive method for the diagnosis of cardiac chamber enlargement [3]. However, the use of a 12-lead ECG can help in the detection of cardiac chamber enlargement in the absence of an echocardiographic examination. Indeed, abnormalities in the amplitude, duration, and shape of the ECG waves have been proposed as indirect signs of pathological changes, such as hypertrophy and/or dilation of cardiac chambers.

Conventional lead placement for the recording of 12-lead ECG has been established in human medicine, and several electrocardiographic criteria for cardiac enlargement have been described, such as: R/S ratio in lead V1, V5, and V6; S wave amplitude in lead V5 and V6 [2,4,5,6,7,8]. Various electrocardiographic criteria for ventricular enlargement have also been suggested in veterinary medicine, such as: S wave amplitude in lead I and lead II; Q wave amplitude in lead aVR; the mean electrical axis (MEA) of the QRS complex on the frontal plane; S wave amplitude in V2 and V4; R/S ratio in lead V4 [9,10,11,12]. However, the number of electrocardiographic criteria validated for the diagnosis of ventricular enlargement in dogs is fewer than those used in humans. Furthermore, most of the proposed criteria have been validated using postmortem examination or chest thoracic radiograph as gold standards for evaluating ventricular enlargement, when transthoracic echocardiography is nowadays the most used diagnostic tool to assess cardiac enlargement in clinical practice. In addition, various precordial lead systems have been used in dogs; hence, the different electrode placement has determined a considerable variability in the amplitude of the recorded electrocardiographic waves yielding significant differences when comparing study results.

One of the most used precordial lead systems in dogs is that one described by Kraus et al. [13]. It has been recently modified by Santilli et al. [14], aiming to obtain more accurate recordings of right atrial and right ventricular depolarization in healthy dogs with various thoracic conformations (i.e., brachymorphic, mesomorphic, and dolichomorphic morphotypes). The main variation of this novel lead system is the different position of V1, which is placed at the costo-chondral junction of the right first intercostal space, while the remaining leads (V2-V6) are placed in the same location previously proposed by Kraus et al. (i.e., the left sixth intercostal space). However, the diagnostic accuracy of this novel precordial lead system has not yet been evaluated in terms of its capacity to identify right ventricular enlargement (RVE) in dogs.

Therefore, the aim of the present study was to evaluate if this novel precordial lead system may increase the diagnostic accuracy of 12-lead ECG in comparison to standard 6-lead ECG for the detection of echocardiographic RVE in dogs.

## 2. Materials and Methods

### 2.1. Animals

The protocol of the study was examined and approved by the Institutional Welfare and Ethics Committee of the University of Pisa (authorization number 52/2020). Client-owned dogs referred for a cardiologic evaluation at the Veterinary Teaching Hospital of the Department of Veterinary Sciences of the University of Pisa and at Anicura Istituto Veterinario Novara were enrolled in this prospective and observational study. Dogs were eligible for participation in the study provided that the owner had given informed consent. A total of 84 dogs of different breeds were included, of which 39 were females (46%), and 45 were males (54%). There were 12 mixed breed dogs, 10 Labrador Retrievers, 8 Chihuahuas, 8 French Bulldogs, 6 Golden Retrievers, 4 Boxers, 3 English Bulldogs; the remaining 33 dogs belonged to 22 different breeds. All dogs enrolled in the study underwent complete physical examination, 12-lead ECG examination, and transthoracic echocardiography. Dogs presenting pleural and/or pericardial effusion were excluded from the study. Data regarding age, sex, body weight (BW), ECG, and echocardiographic findings were collected.

### 2.2. Echocardiography

All echocardiographic examinations were performed by a board-certified cardiologist or by residents supervised by a board-certified cardiologist using ultrasound systems. Complete transthoracic 2D, M-mode, and Doppler echocardiographic examinations were achieved following standard recommendations [15]. Each dog was examined under gentle manual restraint, in the right and left lateral recumbency, without sedation. Right ventricular enlargement was defined using two-dimensional echocardiography as follows: right ventricular free wall thickness normalized for BW (RVFWn) > 0.39 cm/kg^0.250^ [16] and/or a right ventricular end-diastolic area normalized for BW (RVEDAn) > 1.4 cm^2^/kg^0.665^ [17]. In detail, the right ventricular free wall thickness was acquired from the right parasternal long axis view, using a trailing edge to trailing edge technique as perpendicular as possible to the long axis of the ventricle from points estimated to be at the mid-ventricular level. The right ventricular end-diastolic area was measured by tracing the endocardial perimeter from the parietal tricuspid annulus along the right ventricular free wall to the right ventricular apex and back to the septal tricuspid annulus along the interventricular septum.

### 2.3. Electrocardiography

Electrocardiographic examinations were recorded with the dogs positioned in right lateral recumbency using electrocardiographic units (MAC 800 and MAC 1600 ECG systems, General Electric Healthcare, Boston, MA, USA) with a sampling frequency of 1000 Hz for acquisition, a 100 Hz low-pass filter and a 0.3–0.5 Hz high-pass filter to decrease respiratory artifact [18]. Limb leads (I, II, III, aVR, aVL, and aVF) were recorded according to the standard technique [19], and precordial leads (V1, V2, V3, V4, V5, V6) were recorded according to Kraus et al. modified by Santilli et al. [13,14].

Lead V1 was placed at the costo-chondral junction of the right first intercostal space, while the sixth intercostal space was used for all the other precordial leads: V2 was placed adjacent to the sternum, V3 was placed midway between V2 and V4, V4 was placed at the costo-chondral junction, and V5 and V6 were sequentially placed dorsal to V4 at a distance equal to that between V3 and V4.

In accordance with human and veterinary literature [2,4,5,6,7,8,9,10,11,12], the following electrocardiographic measurements were assessed on both limb leads and precordial leads: Q wave amplitude (mV); R wave amplitude (mV); S wave amplitude (mV); and R/S wave ratio. The R/S ratio was calculated for all dogs in which the S wave was present. In those cases in which the S wave was not present, the ratio was not evaluated. The algebraic sum of the QRS complex in V1 was calculated [20]. The MEA of the QRS complex expressed in degrees (°) on the frontal plane was calculated using the isoelectric method [21,22]. A right shift deviation of the MEA of the QRS complex was considered for values between +100° and −80° [11,19,23,24].

### 2.4. Statistical Analysis

Descriptive statistics were generated. Electrocardiographic and echocardiographic data were reported as median and range. The receiver operating characteristic (ROC) curve analysis and the Youden index were assessed in order to define the best cutoff for the detection of RVE. Among the ECG parameters evaluated in the study (i.e., Q wave amplitude, R wave amplitude, S wave amplitude, and R/S wave ratio on limb leads and precordial leads), those criteria with an area under the curve (AUC) > 0.7 [25] and specificity (Sp) higher than 90% were considered as having suitable diagnostic accuracy for the detection of RVE. Statistical analysis was performed with Prism 5 (GraphPad Software Inc., San Diego, CA, USA). A value of *p* < 0.05 was considered statistically significant.

## 3. Results

Among the 84 dogs enrolled in the study sample, 57 dogs were healthy and composed the control group, whereas 27 dogs presented RVE. The median age of the control group was 3 years (range: 5 months–13 years) and 5 years (range: 3 months–17 years) for those with RVE. The median BW was 23.7 kg (range: 2.2–53 kg) in the control group and 13 kg (range: 2.6–50.2 kg) in the dogs with RVE. Of those with RVE, 13 dogs had pulmonary valve stenosis, 11 dogs had precapillary pulmonary hypertension, one had an atrial septal defect, one had tricuspid valve dysplasia, and one dog presented both pulmonary valve stenosis and tricuspid valve dysplasia.

Echocardiographic data of the control group and dogs with RVE are reported in Table 1. Among dogs presenting RVE, 9 out of 27 (33%) presented right ventricular free wall thickening only, 11 out of 27 dogs (41%) presented right ventricular dilation only, while the remaining 7 dogs (26%) presented both right ventricular free wall thickening and right ventricular dilation.

On limb leads, only Q wave amplitude in aVR and the presence of right shift of the MEA of the QRS complex showed suitable diagnostic accuracy for the detection of RVE (Table 2). Among dogs with RVE, 12 out of 27 (44%) showed at least a Q wave amplitude in aVR > 0.1 mV or right shift of the MEA of the QRS complex.

**Figure 1:**
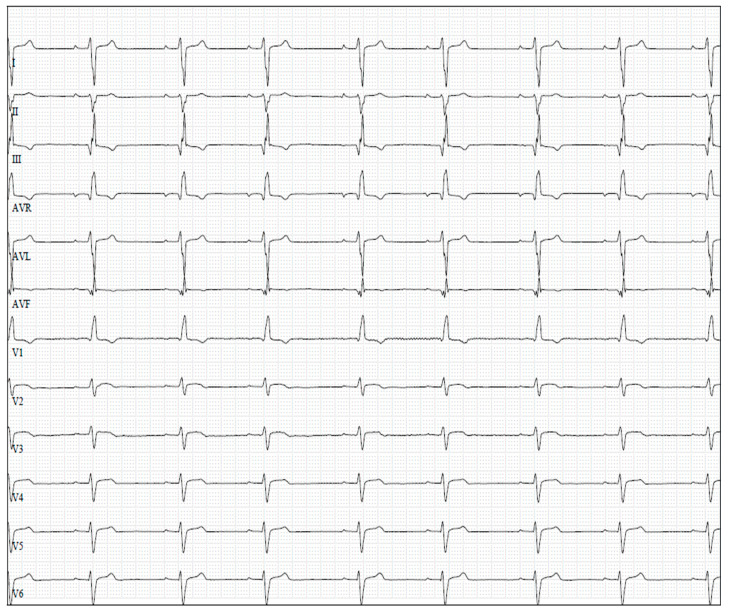
Limb leads and precordial leads of an English Bulldog with pulmonary valve stenosis and right ventricular enlargement showing a Q wave > 0.1 mV in aVR, right shift of MEA, R/S wave ratio < 1.15 in V4, R/S wave ratio < 1.95 in V5 and S wave > 0.7 mV in V6. Paper speed = 50 mm/s; 5 mm/mV.

On precordial leads, the R/S ratio in lead V4, R/S in V5, and S wave amplitude in lead V6 showed suitable diagnostic accuracy for the detection of RVE (Table 2). In 2 out of 27 dogs with RVE in our study, the R/S ratio in V4 was not assessable due to the absence of an S wave on V4. Similarly, in 3 out of 27 dogs with RVE, the R/S ratio in V5 was not assessable because of the absence of an S wave on V5. Among dogs with RVE, 19 out of 27 (70%) showed at least one of the following precordial lead criteria: R/S ratio in V4 < 1.15, R/S ratio in V5 < 1.95, or S wave in V6 > 0.7 mV (Figure 1). Conversely, only 2 out of 27 dogs with RVE (7%) did not have any of the limb or precordial leads criteria for RVE identified in this study.

## 4. Discussion

The present study aimed to evaluate the diagnostic accuracy of 12-lead ECG for the diagnosis of RVE using the precordial lead system proposed by Kraus et al. and modified by Santilli et al. and compared it with the diagnostic efficacy of standard 6-lead ECG for the same purpose.

Considering limb leads, the amplitude of the Q wave in aVR was the only ECG measurement that showed suitable diagnostic accuracy for the detection of RVE. The diagnostic accuracy of the Q wave in aVR for the detection of RVE has already been described in dogs [11,23,24]; however, a different diagnostic cutoff was previously reported (i.e., >0.3 mV in comparison to >0.1 mV found in the present study). This could be explained by different study samples, with different types and severities of cardiac diseases causing RVE, as well as the different thoracic morphotypes of the dogs included. Regarding limb leads, also the amplitude of the S wave in leads I and II were previously described as diagnostic criteria for RVE in dogs [11,19,23,24]; however, they did not match the criteria of the present study for the definition of suitable diagnostic accuracy.

Finally, the MEA of the QRS complex on the frontal plane was evaluated on limb leads that showed suitable diagnostic accuracy for the detection of RVE in dogs, in line with human and veterinary literature [11,12,19,26,27,28]. Specifically, 9 out of 27 dogs with RVE (33%) had a right shift of the MEA, showing high specificity (95%) but low sensitivity for the detection of RVE. Among those nine dogs, eight of them presented RV concentric hypertrophy secondary to pulmonary valve stenosis, while the remaining dog presented RV eccentric hypertrophy secondary to severe pulmonary hypertension of precapillary origin. The low sensitivity of the 6-lead ECG for detecting RVE has also been described in humans, especially when RVE is associated with chronic lung disease, acquired heart disease, and primary pulmonary hypertension, usually characterized by RV eccentric or mixed hypertrophy [2]. These findings may suggest that RV concentric hypertrophy more frequently determines the right shift of the MEA of the QRS complex in comparison to RV eccentric hypertrophy. In line with this hypothesis, electrocardiography showed its best diagnostic accuracy for the detection of RVE in infants with congenital heart disease, usually characterized by RV concentric hypertrophy [2], and a recent study in dogs showed that the degree of right shift of MEA of the QRS complex is predictive of the severity of pulmonary valve stenosis [29]. Therefore, our results could suggest that 6-lead ECG could play a complementary role in the detection of RVE, particularly useful in those cases presenting severe RV concentric hypertrophy rather than in those with RV eccentric hypertrophy. However, further prospective studies aiming to detect the electrocardiographic differences between RV concentric hypertrophy versus RV eccentric hypertrophy in a wider sample of dogs are needed to verify our preliminary findings.

Regarding precordial leads, the R/S ratio in V4, the R/S ratio in V5, and the amplitude of the S wave in V6 showed suitable diagnostic accuracy in distinguishing dogs with RVE and healthy dogs. From an electrophysiological point of view, ventricular depolarization is composed of 3 vectors: the first vector represents the septal depolarization, and it determines a first positive deflection in V1 and a negative deflection in V5 and V6, while the second and the third vectors determine a negative deflection in V1 and positive deflections from V2 to V6. Accordingly, the typical QRS morphology identified in humans and in dogs with normal cardiac dimensions by using precordial leads is characterized by a low-amplitude R wave and deep S wave in V1 (i.e., R/S < 1) and a higher R wave and a lower S wave in lead V2 to V6. [14,30]. Differently, in cases of RVE, there is a modification of the balance between right and left ventricular depolarization vectors as right ventricular depolarization predominates over left ventricular depolarization. Accordingly, the QRS morphology is usually characterized by a higher R wave and lower S wave in V1 and by a lower R wave and higher S wave in V2 to V6 [2,4].

In the present study, the value of the R/S ratio in V4 (cutoff < 1.15 mV) and its specificity (96%) described in our study were similar to those previously reported in the veterinary literature: R/S ratio in V4 < 1 mV with specificity 96% [12] and R/S ratio in V4 < 0.87 mV with specificity of 98% [11,19,23,24]. Differently, our cutoff of the R/S ratio in V4 showed a higher sensitivity than in previous studies (63% versus 37%). It is noted that in 2 out of 27 dogs with RVE in our study, the R/S ratio in V4 was not assessable due to the absence of an S wave on V4 in these cases.

To the best of the author’s knowledge, the diagnostic value of the R/S ratio in V5 and the S wave amplitude in V6 has never been described for the detection of RVE in dogs. On the other hand, these criteria are used in human cardiology for the detection of RVE [5,6]. Interestingly, the reported cutoff of the S wave amplitude in V6 for humans [5,6] was identical to that found in our study (>0.7 mV), whereas the cutoff of the R/S ratio in V5 described in our study was almost half of that reported in the human literature [5]. Similar to the R/S ratio in V4, in 3 out of 27 dogs with RVE, the R/S ratio in V5 was not assessable because of the absence of an S wave on this lead. Differently, The diagnostic accuracy of the R/S ratio and S wave amplitude on the remaining leads was not suitable.

Our results are in line with previous studies describing the diagnostic accuracy of electrocardiography as having low to moderate sensitivity but high specificity in detecting RVE [2,5,6]. Among our study sample, 44% of dogs with RVE showed at least one of the identified limb lead criteria for RVE (Q wave in aVR or MEA of the QRS complex). On the other hand, considering precordial leads, the percentage of dogs presenting at least one of the identified electrocardiographic criteria for RVE increased to 70% (19 out of 27 dogs). Based on these findings, our results may suggest that the assessment of precordial leads may be beneficial for increasing the diagnostic accuracy of ECG for the detection of RVE.

In the novel precordial lead system proposed by Santilli et al. [14], V1 was shifted from the fifth right intercostal space originally proposed by Kraus et al. [13] to the first right intercostal space with the aim of obtaining a more accurate recording of right atrial and right ventricular depolarization. Accordingly, no previous studies dealt with the accuracy and the assessment of precordial leads according to the different thoracic morphotypes. To the results of that study, the old V1 placement at the level of the fifth intercostal space yielded an unreliable right ventricular electrocardiographic pattern in dogs with various thoracic conformations [14].

Despite the novel precordial lead placement, in the present study, V1 did not show suitable diagnostic accuracy for the detection of RVE in dogs. However, when the algebraic sum of the QRS complex in V1 was evaluated, 9 out of 27 dogs with RVE (33%) showed a positive QRS complex, of which 7 dogs presented RV concentric hypertrophy secondary to moderate to severe pulmonic stenosis, while the remaining 2 dogs presented RV eccentric hypertrophy secondary to an atrial septal defect in one case and severe tricuspid valve dysplasia in the other one. Thus, it could be suggested that RV concentric hypertrophy could lead to a positive morphology of the QRS in V1 rather than RV eccentric hypertrophy in the system proposed by Santilli et al. However, given the small sample size of dogs presenting RVE, the present study was probably underpowered to identify a possible diagnostic criterion for detecting RVE in V1. Further prospective studies evaluating the diagnostic performance of lead V1 for the detection of RVE are needed, especially comparing different types of right ventricular remodeling and different thoracic morphotypes.

The clinical usefulness of the present study is represented by the potential usage of 12-lead ECG in dogs presented with cardio-respiratory symptoms in a first opinion practice (such as syncope, exercise intolerance, respiratory distress, ascites) or in asymptomatic dogs during a pre-anesthetic ECG evaluation. The assessment of a 12-lead ECG may allow general practitioners and anesthesiologists to perform a preliminary cardiologic evaluation, possibly suggesting right heart cardiomegaly. Indeed, ECG is a common diagnostic tool that is easy to assess, less cost-effective, less experience-dependent, and easy to share in telemedicine in comparison to echocardiography. Based on the results of the present study, the finding of one of the described 12-lead ECG criteria could constitute a concrete clinical suggestion of underlying right heart disease, strongly suggesting the need for a specialistic echocardiographic examination.

The results of the present study must be carefully considered in the context of its limitations. At first, a low number of dogs with RVE was included. Further studies comparing 12-lead ECG findings in a larger number of dogs with RVE secondary to different etiologies and severity are needed. At second, age, BW, and type of thoracic morphotype were not statistically matched between the control group and dogs with RVE. However, large and small breed dogs were included in the control and dogs with RVE groups. Similarly, brachycephalic dogs were present in both groups. Third, only two-dimensional echocardiographic parameters of right ventricular size (right ventricular free wall thickness and right ventricular end-diastolic area) were evaluated in the present study. Nevertheless, RV volume has been proposed as a possibly more reliable method for the non-invasive assessment of RV dimension [17,31,32]. Moreover, there are currently no consensus guidelines in veterinary medicine dealing with the objective assessment of right heart size in dogs. Finally, the precordial ECG criteria identified in the present study were not matched with the severity of RVE. Different from human medicine [32], there are currently no consensus guidelines in veterinary medicine dealing with the objective assessment of right heart size and the severity classification of RVE in dogs. Thus, further prospective studies evaluating the reliability of the reported precordial criteria to the objective severity of RVE are warranted.

## 5. Conclusions

The 12-lead ECG using the precordial lead system of Kraus et al. modified by Santilli et al. has low sensitivity but high specificity for the diagnosis of RVE in dogs. The R/S ratio in V4 and V5 and the S wave amplitude in V6 showed the best diagnostic accuracy in distinguishing between dogs presenting RVE and healthy dogs. Adding these precordial lead criteria to the standard 6-lead ECG evaluation increases the diagnostic accuracy of RVE detection in dogs. Further studies evaluating the electrocardiographic differences among dogs with concentric versus eccentric RV hypertrophy would be of clinical value.

## Figures and Tables

**Table 1:** Echocardiographic values in the study sample (n = 84 dogs).

	Control Dogs (n = 57)	Dogs with RVE (n = 27)
**RVFWn**	0.20 (0.15–0.27)	0.38 (0.19–0.64)
**RVEDAn**	0.83 (0.57–1.19)	1.36 (0.74–4.51)

Abbreviations: RVE, right ventricular enlargement; RVFWn, right ventricular free wall thickness normalized for body weight; RVEDAn, right ventricular end-diastolic area normalized for body weight. Data are reported as median (range).

**Table 2:** Electrocardiographic parameters showing suitable diagnostic accuracy in the detection of right ventricular enlargement.

	Control Dogs	Dogs with RVE	Cutoff	Se (%)	Sp (%)	AUC
**Limb leads**						
Q wave-aVR (mV)	0 (0–1.1)	0.2 (0–2.2) *	>0.10	53	95	0.727
MEA of QRS (°)	74° (15–105°)	75° (−105–165°)	Right shift	33	95	NA
**Precordial leads**						
R/S ratio-V4	4.5 (0.75–34)	1.1 (0.18–11.3) *	<1.15	63	96	0.842
R/S ratio-V5	5 (0.33–18.5)	1 (0.1–26) *	<1.95	69	92	0.839
S wave-V6 (mV)	0.2 (0–1.8)	0.7 (0–2.8) *	>0.70	52	92	0.703

Abbreviations: AUC, area under the curve; NA, not available; MEA, mean electrical axis; RVE, right ventricular enlargement; Se, sensitivity; Sp, specificity. Data are reported as median (range). * *p* < 0.05 in comparison to the control group.

## Data Availability

The data presented in this study have not been published elsewhere, but are available on request from the corresponding author.
